# Supplementary material for: Resolving the full spectrum of human genome variation using Linked-Reads
Source: Genome Res. 2019 Apr;29(4):635–45. doi: 10.1101/gr.234443.118 (PMC6442396; doi:10.1101/gr.234443.118)
Supplement: Supplemental Material [file supp_gr.234443.118_Supplemental_longranger-2.2.zip › longranger-2.2.tar.bz2!/lr2.2-public/lib/stan/cmdstan/stan/doc/api/html/fvar_8hpp_source.html]

Stan: src/stan/agrad/fwd/fvar.hpp Source File


|  |  |
| --- | --- |
|  | Stan  2.5.0  probability, sampling & optimization |


- Main Page
- Related Pages
- Namespaces
- Classes
- Files

- File List
- File Members

All Classes Namespaces Files Functions Variables Typedefs Enumerations Enumerator Friends Macros Pages

- src
- stan
- agrad
- fwd

fvar.hpp

Go to the documentation of this file.

1 #ifndef STAN\_\_AGRAD\_\_FWD\_\_FVAR\_\_HPP

2 #define STAN\_\_AGRAD\_\_FWD\_\_FVAR\_\_HPP

3

4 #include <ostream>

5 #include <boost/math/special\_functions/fpclassify.hpp>

6 #include <stan/meta/likely.hpp>

7

8 namespace stan {

9

10  namespace agrad {

11

12  template <typename T>

13  struct fvar {

14

15  T val\_; // value

16  T d\_; // tangent (aka derivative)

17

18  T val() { return val\_; }

19  T tangent() { return d\_; }

20

21  typedef fvar value\_type;

22

23  // TV and TD must be assignable to T

24  template <typename TV, typename TD>

25  fvar(const TV& val, const TD& deriv) : val\_(val), d\_(deriv) {

26  if (unlikely(boost::math::isnan(val)))

27  d\_ = val;

28  }

29

30  // TV must be assignable to T

31  template <typename TV>

32  fvar(const TV& val) : val\_(val), d\_(0.0) {

33  if (unlikely(boost::math::isnan(val)))

34  d\_ = val;

35  }

36

37  fvar() : val\_(0.0), d\_(0.0) { }

38

39  template <typename T2>

40  inline

41  fvar<T>&

42  operator+=(const fvar<T2>& x2) {

43  val\_ += x2.val\_;

44  d\_ += x2.d\_;

45  return \*this;

46  }

47

48  template <typename T2>

49  inline

50  fvar<T>&

51  operator+=(const T2& x2) {

52  val\_ += x2;

53  return \*this;

54  }

55

56  template <typename T2>

57  inline

58  fvar<T>&

59  operator-=(const fvar<T2>& x2) {

60  val\_ -= x2.val\_;

61  d\_ -= x2.d\_;

62  return \*this;

63  }

64

65  template <typename T2>

66  inline

67  fvar<T>&

68  operator-=(const T2& x2) {

69  val\_ -= x2;

70  return \*this;

71  }

72

73  template <typename T2>

74  inline

75  fvar<T>&

76  operator\*=(const fvar<T2>& x2) {

77  d\_ = d\_ \* x2.val\_ + val\_ \* x2.d\_;

78  val\_ \*= x2.val\_;

79  return \*this;

80  }

81

82  template <typename T2>

83  inline

84  fvar<T>&

85  operator\*=(const T2& x2) {

86  val\_ \*= x2;

87  return \*this;

88  }

89

90  // SPEEDUP: specialize for T2 == var with d\_ function

91

92  template <typename T2>

93  inline

94  fvar<T>&

95  operator/=(const fvar<T2>& x2) {

96  d\_ = (d\_ \* x2.val\_ - val\_ \* x2.d\_) / ( x2.val\_ \* x2.val\_);

97  val\_ /= x2.val\_;

98  return \*this;

99  }

100

101  template <typename T2>

102  inline

103  fvar<T>&

104  operator/=(const T2& x2) {

105  val\_ /= x2;

106  return \*this;

107  }

108

109  inline

110  fvar<T>&

111  operator++() {

112  ++val\_;

113  return \*this;

114  }

115

116  inline

117  fvar<T>

118  operator++(int /\*dummy\*/) {

119  fvar<T> result(val\_,d\_);

120  ++val\_;

121  return result;

122  }

123

124  inline

125  fvar<T>&

126  operator--() {

127  --val\_;

128  return \*this;

129  }

130  inline

131  fvar<T>

132  operator--(int /\*dummy\*/) {

133  fvar<T> result(val\_,d\_);

134  --val\_;

135  return result;

136  }

137

138  friend

139  std::ostream&

140  operator<<(std::ostream& os, const fvar<T>& v) {

141  return os << v.val\_ << ':' << v.d\_;

142  }

143  };

144  }

145 }

146 #endif

boost::math::isnan

bool isnan(const stan::agrad::var &v)

Checks if the given number is NaN.

**Definition:** boost\_fpclassify.hpp:69

stan::agrad::fvar::fvar

fvar()

**Definition:** fvar.hpp:37

stan::agrad::fvar::operator/=

fvar< T > & operator/=(const fvar< T2 > &x2)

**Definition:** fvar.hpp:95

stan::agrad::fvar::operator--

fvar< T > & operator--()

**Definition:** fvar.hpp:126

stan::agrad::fvar

**Definition:** fvar.hpp:13

stan::agrad::fvar::operator++

fvar< T > operator++(int)

**Definition:** fvar.hpp:118

stan::agrad::fvar::operator++

fvar< T > & operator++()

**Definition:** fvar.hpp:111

stan::agrad::fvar::operator-=

fvar< T > & operator-=(const fvar< T2 > &x2)

**Definition:** fvar.hpp:59

stan::agrad::fvar::val

T val()

**Definition:** fvar.hpp:18

unlikely

#define unlikely(x)

**Definition:** likely.hpp:9

stan::agrad::fvar::operator+=

fvar< T > & operator+=(const T2 &x2)

**Definition:** fvar.hpp:51

stan::agrad::fvar::operator/=

fvar< T > & operator/=(const T2 &x2)

**Definition:** fvar.hpp:104

stan::agrad::fvar::operator--

fvar< T > operator--(int)

**Definition:** fvar.hpp:132

stan::agrad::fvar::value\_type

fvar value\_type

**Definition:** fvar.hpp:21

likely.hpp

stan::agrad::fvar::fvar

fvar(const TV &val, const TD &deriv)

**Definition:** fvar.hpp:25

stan::agrad::fvar::operator-=

fvar< T > & operator-=(const T2 &x2)

**Definition:** fvar.hpp:68

stan::agrad::fvar::operator\*=

fvar< T > & operator\*=(const fvar< T2 > &x2)

**Definition:** fvar.hpp:76

stan::agrad::fvar::operator\*=

fvar< T > & operator\*=(const T2 &x2)

**Definition:** fvar.hpp:85

stan::agrad::fvar::d\_

T d\_

**Definition:** fvar.hpp:16

stan::agrad::fvar::operator+=

fvar< T > & operator+=(const fvar< T2 > &x2)

**Definition:** fvar.hpp:42

stan::agrad::fvar::tangent

T tangent()

**Definition:** fvar.hpp:19

stan::agrad::fvar::fvar

fvar(const TV &val)

**Definition:** fvar.hpp:32

stan::agrad::fvar::val\_

T val\_

**Definition:** fvar.hpp:15


---

[ Stan Home Page ]

*© 2011–2014,
Stan Development Team.*
